# Control variables of serum ferritin concentrations in hospitalized newborn infants: an observational study

**DOI:** 10.1038/s41598-023-35404-0

**Published:** 2023-05-24

**Authors:** Tadashi Hisano, Junichiro Okada, Kennosuke Tsuda, Sachiko Iwata, Shinji Saitoh, Osuke Iwata

**Affiliations:** 1 Center for Human Development and Family Science, Department of Neonatology and Pediatrics, Nagoya City University Graduate School of Medical Sciences, Nagoya, Aichi, 467-8601 Japan; 2 Division of Neonatology, St. Mary’s Hospital, Fukuoka, Japan

**Keywords:** Medical research, Paediatric research

## Abstract

Both iron excess and deficiency are deleterious to cellular and organ homeostasis. Serum ferritin levels serve as a biomarker of iron storage; however, their distribution and determinants in sick newborn infants remain unclear. This study aimed to investigate the reference range and independent variables of serum ferritin in hospitalized newborn infants. All newborn infants who were hospitalized at a tertiary neonatal center within 24 h of birth were retrospectively reviewed for the period of April 2015 through March 2017. Serum ferritin levels were assessed using venous blood samples obtained at admission and their independent variables were explored. The study population comprised 368 infants (36.2 ± 2.8 weeks gestation and 2319 ± 623 g at birth), whose median serum ferritin level was 149 µg/L (inter-quartile range: 81–236). The multivariable model used to explain serum ferritin values comprised hemoglobin, lactate dehydrogenase, blood pH, and maternal hypertensive disorders in pregnancy (all *p <* 0.01, adjusted for sex and birth weight). Serum ferritin values in hospitalized newborn infants were comparable to those previously reported using umbilical cord blood. Our novel findings indicated the association between blood pH, lactate dehydrogenase, and ferritin levels, suggesting the influence of antenatal hypoxia–ischemia and stress to serum ferritin levels.

## Introduction

Iron is a trace metal that plays an essential role in various biological processes^1^. It transports oxygen as hemoglobin in blood circulation, exchanges electrons easily in oxidation–reduction reactions in organs, and is essential for synthesis of adenosine triphosphates by oxidative phosphorylation in the mitochondria^1,2^. Iron is also necessary for deoxyribonucleic acid synthesis^1,3–5^. Deficiency and excess of iron in newborn infants can cause various pathological conditions^6–8^.

The maintenance of iron homeostasis involves various factors, such as erythropoiesis, cellular iron transportation, and iron storage^9,10^. Iron regulation in demand, storage, and erythropoiesis can be described using the integrity of the erythropoietin-erythroferrone-hepcidin axis^11^. Serum ferritin levels have been used as a marker of iron excess and deficiency^12,13^. The ferritin protein consists of H and L subunits^14,15^. The H subunit of ferritin converts excessive intracellular ferric ion (Fe^2+^) into ferrous iron (Fe^3+^), whereas the L subunit has a central structure that stores ferrous iron (Fe^3+^) within the cell. Although only a part of the L subunit appears in the blood, serum ferritin levels reflect the amount of ferritin within the whole body^16^. Ferritin production is controlled at the translation level of the ferritin gene, with the feedback triggered by the excess and shortage of iron^17^.

Fetal and neonatal iron deficiency and subsequent dysfunctions in adenosine triphosphates, deoxyribonucleic acid synthesis, and myelination may lead to increased incidence of death and neurodevelopmental disorders^18–20^. In newborn infants, serum ferritin levels are positively associated with gestational age and birth weight, whereas placental dysfunction is associated with low ferritin levels, and elevated ferritin levels are observed in association with infection, inflammation, and neurodegenerative diseases^21–26^. Marked elevation of ferritin levels is observed in relatively rare clinical conditions, such as human herpes simplex infection, hemophagocytic lymphohistiocytosis, and neonatal hemochromatosis in newborn infants^27–29^. Bronchopulmonary dysplasia, retinopathy of prematurity, and necrotizing enterocolitis are relatively common diseases in hospitalized newborn infants^30^. Altered iron metabolism, as well as inflammation and oxygen radical formation, is considered to be involved within an important part of the disease mechanism^23,31^. Although these clinical conditions cause a wide range of morbidities^32^, the timing and level of altered iron metabolism and related ferritin levels largely remain unknown.

Adverse consequences related to impaired iron metabolism might be identified and ameliorated by monitoring ferritin levels shortly after birth. However, thus far, reference ranges of serum ferritin levels in newborn infants have not been established based on a large sample size. Reference ranges of serum ferritin levels are ethically difficult to establish in healthy newborn infants. However, in hospitalized newborn infants, serum ferritin levels can be assessed as a routine or additional test using residual blood samples. If the reference ranges can be established in hospitalized infants, they may help design and conduct future studies to explore covert relationships between altered iron metabolism and pathological clinical conditions. In addition, despite the dynamic change in serum ferritin levels in newborn infants under both physiological and pathological conditions, clinical factors associated with ferritin levels in newborn infants remain largely unknown.

This study aimed to clarify the reference range of serum ferritin levels in a relatively large cohort of hospitalized newborn infants and to elucidate their independent variables.

## Results

Values are presented as mean ± standard deviation unless otherwise described.

### Clinical backgrounds

This study cohort included 368 newborn infants of 36.2 ± 2.8 weeks gestation and 2319 ± 623 g at birth (Table 1) who were hospitalized due to preterm birth and/or low birth weight (n = 186), respiratory failure (n = 86), birth asphyxia (n = 18), infection (n = 13), hypoglycemia (n = 10), cardiac disease (n = 9), minor anomalies (n = 9), chromosomal abnormalities (n = 8), gastrointestinal symptoms (n = 7), jaundice (n = 1), or other reasons (n = 21). Four neonates died due to severe congenital cardiac disease (n = 2), systemic herpes simplex infection (n = 1), and hypoxic-ischemic encephalopathy (n = 1) before discharge.

**Table 1:** Background characteristics of included patients.

Variables	
Maternal information	
Age (year)	31.4 ± 5.6
Primipara	133 (36.1)
Multiple pregnancy	63 (17.1)
Gestational diabetes mellitus	14 (3.8)
Hypertensive disorders in pregnancy	46 (12.5)
Maternal transport	128 (34.8)
Antenatal steroid	69 (18.8)
Preterm rupture of membranes	86 (23.4)
Meconium-stained amniotic fluid	41 (11.1)
Cesarean delivery	187 (50.8)
Variables at birth	
Male sex	206 (56.0)
Gestational age at birth (week)	36.2 ± 2.8
Body weight (g)	2319 ± 623
Body height (cm)	44.6 ± 3.9
Head circumference (cm)	31.7 ± 2.2
Apgar score at 1 min	8 [7, 8]
Apgar score at 5 min	9 [8, 9]
Small for gestational age	78 (21.2)
Large for gestational age	7 (1.9)
Inborn infants	258 (70.1)
Blood biomarkers	
Ferritin (μg/L)	149 [81.3, 236]
Hemoglobin (g/dL)	18.2 [16.4, 20.0]
White blood cell count (10^3^/mm^3^)	13.0 [9.8, 17.6]
Monocyte count (10^3^/mm^3^)	6.7 [3.5, 10.6]
Platelet count (10^3^/mm^3^)	2.54 [2.11, 2.98]
Lactate dehydrogenase (IU/L)	448 [388, 589]
C-reactive protein (mg/dL)	0.0 [0.0, 0.0]
pH	7.294 [7.236, 7.341]
Base excess (mEq/L)	-3.4 [-7.0, -1.3]
Clinical comorbidities	
Positive blood culture at admission	0 (0.0)
Symptomatic ductus arteriosus*	12 (3.2)
Symptomatic ductus arteriosus requiring surgical closure	6 (1.6)
Intestinal perforation	1 (0.3)
Respiratory failure requiring mechanical ventilation	118 (32.1)
Prolonged mechanical ventilation > 72 h	71 (19.3)
Chronic lung disease assessed on day 28	1 (0.3)
Severe retinopathy of prematurity requiring photocoagulation	1 (0.3)
Death	4 (1.1)

Values are shown as mean ± standard deviation, number (%), or median [inter-quartile ranges].

*Defined as ductus arteriosus requiring indomethacin (excluding prophylactic) or surgery.

### Serum ferritin values and their independent variables

The serum ferritin level for the whole study cohort of infants was 149 (inter-quartile range [IQR]: 81–236) µg/L (See Online Supplementary Table 1 for values of different gestational age groups).

In the univariable analysis, ferritin levels were positively associated with gestational age (per week; regression coefficient [B], 0.128; 95% confidence interval [CI], 0.089–0.167; *p <* 0.001), body weight (per 1kg; B, 0.662; 95% CI, 0.492–0.833; *p <* 0.001), the z-score of body weight (B, 0.412; 95%CI, 0.306–0.518; *p* <0.001), body height (per cm; B, 0.114; 95%CI, 0.086–0.141; *p* < 0.001), the z-score of body height (B, 0.439; 95%CI, 0.334–0.544; *p* < 0.001), head circumference (per cm; B, 0.177; 95%CI, 0.129–0.225; *p* < 0.001), the z-score of head circumference (B, 0.390; 95%CI, 0.284–0.497; *p* < 0.001), small for gestational age (B, -0.540; 95%CI, -0.813 to -0.267; *p* < 0.001), white blood cell count (per 10^3^/mm^3^; B, 0.034; 95% CI, 0.022–0.047; *p <* 0.001), monocyte  count (per 10^2^/mm^3^; B, 0.039; 95%CI, 0.020–0.059; p < 0.001), C-reactive protein (per mg/dL; B, 0.176; 95% CI, 0.044–0.309; *p =* 0.009), lactate dehydrogenase (per 10^2^ IU/L; B, 0.102; 95% CI, 0.078–0.126; *p <* 0.001), and meconium-stained amniotic fluid (B, 0.511; 95% CI, 0.152–0.869; *p =* 0.005). Inverse associations with the ferritin levels were observed for hemoglobin levels (per g/dL; B, - 0.119; 95% CI, - 0.157 to - 0.080; *p <* 0.001), Apgar scores at 1 min (B, -0.132; 95% CI, -0.185 to -0.079; *p <* 0.001) and 5 min (B, -0.208; 95% CI: -0.290 to -0.126; *p <* 0.001), blood pH (B, -2.679; 95% CI, -3.744 to -1.614; *p <* 0.001) and blood base excess (per mEq/L; B, -0.082; 95% CI, -0.107 to -0.057; *p <* 0.001), inborn (B, -0.746; 95% CI, -0.983 to -0.509; *p <* 0.001), maternal age (per year; B, -0.027; 95% CI, -0.048 to -0.007; *p =* 0.008), hypertensive disorders in pregnancy (B, -0.768; 95% CI, -1.104 to -0.432, *p <* 0.001), multiple pregnancy (B, -0.444; 95% CI, -0.743 to -0.144; *p =* 0.004), respiratory failure requiring mechanical ventilation (B, -0.252; 95%CI, -0.495 to -0.009, *p* = 0.042), and prolonged mechanical ventilation > 72 h (B, -0.436; 95% CI, -0.721 to -0.150; *p =* 0.003) (Table 2).

**Table 2:** Findings from univariable analysis.

Variables	Mean	Regression coefficient		*P*-value
		95% confidence interval		
		Lower	Upper	
Maternal information				
Maternal age (year)	-0.027	-0.048	-0.007	0.008
Primipara	-0.045	-0.124	0.033	0.259
Multiple pregnancy	-0.444	-0.743	-0.144	0.004
Gestational diabetes mellitus	-0.326	-0.921	0.269	0.282
Hypertensive disorders in pregnancy	-0.768	-1.104	-0.432	< 0.001
Antenatal steroid	-0.201	-0.492	0.091	0.176
Preterm rupture of membrane	0.076	-0.193	0.345	0.579
Meconium-stained amniotic fluid	0.511	0.152	0.869	0.005
Cesarean delivery	-0.111	-0.352	0.130	0.364
Variables at birth				
Male sex	0.077	-0.153	0.306	0.511
Gestational age (week)	0.128	0.089	0.167	< 0.001
Body weight (kg)	0.662	0.492	0.833	< 0.001
Z-score	0.412	0.306	0.518	< 0.001
Body height (cm)	0.114	0.086	0.141	< 0.001
Z-score	0.439	0.334	0.544	< 0.001
Head circumference (cm)	0.177	0.129	0.225	< 0.001
Z-score	0.390	0.284	0.497	< 0.001
Small for gestational age	-0.540	-0.813	-0.267	< 0.001
Large for gestational age	-0.486	-1.308	0.336	0.245
Apgar score at 1 min	-0.132	-0.185	-0.079	< 0.001
Apgar score at 5 min	-0.208	-0.290	-0.126	< 0.001
Inborn infants	-0.746	-0.983	-0.509	< 0.001
Blood biomarkers at admission				
Hemoglobin (g/dL)	-0.119	-0.157	-0.080	< 0.001
White blood cell (10^3^/mm^3^)	0.034	0.022	0.047	< 0.001
Monocyte count (10^2^/mm^3^)	0.039	0.020	0.059	< 0.001
Platelet count (10^5^/mm^3^)	0.078	− 0.069	0.226	0.295
Lactate dehydrogenase (10^2^ IU/L)	0.102	0.078	0.126	< 0.001
C-reactive protein (mg/dL)	0.176	0.044	0.309	0.009
pH	-2.679	-3.744	-1.614	< 0.001
Base excess (mEq/L)	-0.082	-0.107	-0.057	< 0.001
Clinical comorbidities				
Symptomatic ductus arteriosus*	0.262	-0.379	0.903	0.442
Respiratory failure requiring mechanical ventilation	-0.252	-0.495	-0.009	0.042
Prolonged mMechanical ventilation > 72 h	-0.436	-0.721	-0.150	0.003

*Defined as ductus arteriosus requiring indomethacin (excluding prophylactic) or surgery.

The multivariable model used to explain serum ferritin levels comprised the z-score of body weight (B, 0.208; 95% CI, 0.055–0.360; *p =* 0.008), hemoglobin (per g/dL; B, -0.077; 95% CI, -0.111 to -0.042; *p <* 0.001), lactate dehydrogenase (per 10^2^ IU/L; B, 0.076; 95% CI, 0.053–0.098; *p <* 0.001), blood pH (B, -1.363; 95% CI, -2.305 to -0.421; *p =* 0.005), and hypertensive disorders in pregnancy (B, -0.489; 95% CI, -0.779 to -0.198; *p =* 0.001) (Table 3; See Online Supplementary Table 2 for alternative multivariable models).

**Table 3:** Findings from multivariable analysis.

Variables	Mean	Regression coefficient		*P*-value
		95% confidence interval		
		Lower	Upper	
Hypertensive disorders in pregnancy	-0.489	-0.779	-0.198	0.001
Male sex	0.072	-0.122	0.266	0.465
Gestational age (per week)	0.025	-0.029	0.079	0.356
Z-score of body weight	0.208	0.055	0.360	0.008
Blood biomarkers at admission				
Lactate dehydrogenase (per 10^2^ IU/L)	0.076	0.053	0.098	< 0.001
Hemoglobin (per g/dL)	-0.077	-0.111	-0.042	< 0.001
pH	-1.363	-2.305	-0.421	0.005

## Discussion

In a relatively larger population of hospitalized newborn infants, we demonstrated that ferritin levels of venous blood samples assessed shortly after birth were comparable to those measured using umbilical cord blood in term and preterm newborn infants^33^. Consistent with previous studies^33,34^, high serum ferritin levels were observed in newborn infants with greater gestational age and birth weight, and clinical conditions suggestive of placental dysfunction, infection, and inflammation. We newly identified hemoglobin levels, Apgar scores, and blood acid–base status as potential independent variables of ferritin levels. In the multivariable model, higher serum ferritin levels within the first 24 h of age were determined by a greater z-score of the birth weight, lower hemoglobin levels, lower blood pH, higher serum lactate dehydrogenase values, and absence of pregnancy-induced hypertension, suggesting that elevated serum ferritin levels in newborn infants might be associated with fetal growth and maturation, and with acute and chronic perinatal hypoxia–ischemia. With further validation, serum ferritin levels shortly after birth might be used as a predictive biomarker to estimate the impact of hypoxia–ischemia at birth.

Based on a mixed but relatively large population of hospitalized newborn infants, we first defined the reference value of serum ferritin levels. We found that ferritin levels were comparable but slightly lower than 171–200 µg/L, which were reported previously using umbilical cord blood^33,35^. Considering that our study population had a relatively younger mean gestational age of 36.2 weeks as opposed to 41 weeks in the aforementioned study, the slight difference in the ferritin values in these studies might reflect the maturation-related increase in ferritin levels, and our findings might be consistent with the previous understanding that serum ferritin levels increase during the fetal period and exceeds the level of healthy adults by term birth, followed by temporal declines from and recovery to the baseline levels during the first years of life^33,36^.

Using the same dataset, we further delineated clinical independent variables of serum ferritin levels at birth. Consistent with previous reports^33,34^, the gestational age, birth weight, lactate dehydrogenase, and inflammatory biomarkers, including white blood cell count and C-reactive protein, have positive correlations with serum ferritin levels, suggesting that serum ferritin levels at birth are influenced by intrauterine growth and maturation, as well as antenatal infection and inflammation. Similarly, maternal age and the presence of pregnancy-induced hypertension appeared to be inversely correlated with serum ferritin levels.

In addition to established independent variables, we identified several novel control variables of serum ferritin in newborn infants. Regarding the relationship between ferritin and hemoglobin levels in newborn infants, positive and negative relationships have been reported^37,38^. In our study, we demonstrated that ferritin levels were inversely correlated with hemoglobin levels at birth according to a relatively large cohort of hospitalized newborn infants. This finding appears to be controversial considering that iron deficiency theoretically results in reduced serum ferritin and hemoglobin levels^12^. However, prolonged intrauterine hypoxia and ischemia might have upregulated fetal erythropoiesis with lower ferritin levels, similar with placental deficiency and intrauterine growth restriction^26^. Similar inverse relationships between serum ferritin and hemoglobin were reported in association with fetal growth restriction, maternal diabetes mellitus, and maternal smoking^33,39,40^. Since incomplete erythropoiesis leads to improper use of iron and destruction of immature erythrocytes and raises serum ferritin levels under anemic conditions^41^, immature response to erythropoietin in newborn infants might explain the observed inverse correlation between hemoglobin and ferritin levels. In addition, in cases when infection and inflammation coincide with anemia, elevated hepcidin expression may restrict iron supply and erythropoiesis, as shown in a rat model of anemia coincided with chronic inflammation^42^.

We observed increases in ferritin levels in association with clinical events suggestive of the presence of fetal hypoxia–ischemia and acidosis, as well as birth asphyxia, represented by elevated lactate dehydrogenase levels, low blood pH and base excess, and low Apgar scores. These findings are consistent with studies that reported marked elevation of serum ferritin levels after the onset of stroke^43–45^ and with a preclinical study in rodents, which showed that temporary occlusion of the middle cerebral artery resulted in the upregulation of ferritin synthesis at both mRNA and protein levels^44^.

There were several limitations to our current study. First, because the dataset used for the current study was based on the information obtained for clinical reasons, we were not able to assess serum biomarkers of iron metabolism other than ferritin. Future studies may need to assess comprehensive markers involved in iron homeostasis, such as hepcidin, transferrin receptors, erythropoietin, and erythroferrone, as well as serum iron levels. Second, we were unable to recruit healthy newborn infants to establish the reference ranges of ferritin concentrations because of ethical and practical reasons. Consequently, the clinical backgrounds of the hospitalized newborn infants were highly heterogeneous, including extremely preterm birth, hypoxic-ischemic encephalopathy, severe systemic infection, and other clinical conditions, which need to be considered when interpreting our findings to the practice in healthy newborn infants. Such variations in the clinical backgrounds helped identify patient types and clinical conditions associated with ferritin levels shortly after birth. However, despite the moderate size of the study cohort, there were insufficient number of infants with specific diseases, such as septicemia, necrotizing enterocolitis, chronic lung disease, and retinopathy of prematurity, to investigate the relationship between altered iron metabolism and the development of the diseases. Third, although all blood samples were obtained within 24 h of birth, we were unable to incorporate the time of sampling after birth within the model, which might be an additional independent variable of serum ferritin levels considering the drastic changes in oxygen metabolism from intrauterine to the extrauterine environment.

## Conclusions

We clarified a reference value for serum ferritin levels in sick newborns within 24 h of age, which was comparable to previous studies that used cord blood and included healthy adults. We also confirmed the relationship between ferritin levels and established control variables of gestational age, birth weight and its z-score, and those associated with infection and inflammation. Furthermore, hemoglobin levels, Apgar scores, lactate dehydrogenase levels, and blood acid–base status were newly identified as potential independent variables of ferritin levels in newborn infants. Our findings suggested that intrauterine growth and maturation, inflammation, anemia, and acute and chronic hypoxia–ischemia are key factors in determining serum ferritin levels in hospitalized newborn infants.

## Methods

**Figure 1:**
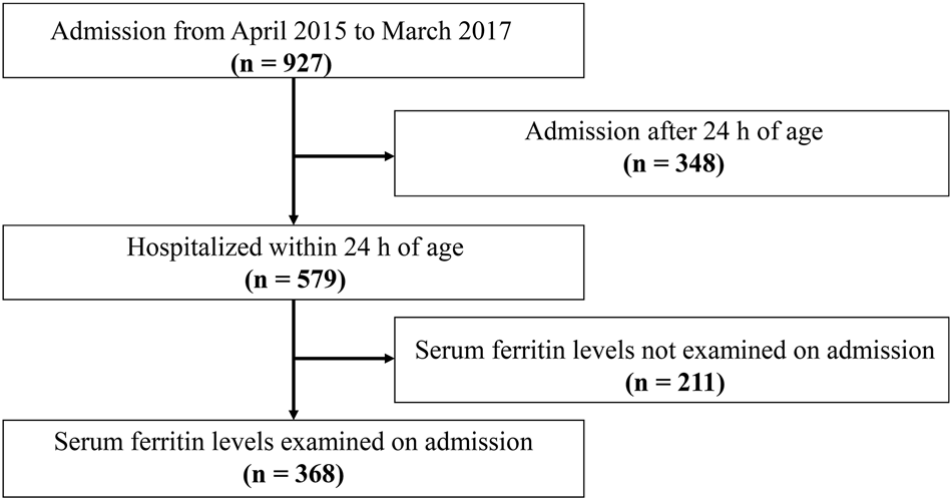
Flow chart of the study population. A diagram depicting the flow of the study population.

This retrospective observational study was conducted in compliance with the Declaration of Helsinki under the approval of the Ethics Committee of St Mary’s Hospital. The Ethics Committee waived the requirement for informed consent because the study only used anonymized data obtained for clinical reasons. Newborn infants who were hospitalized at a tertiary neonatal intensive care unit (NICU) of St Mary’s Hospital within 24 h of age and assessed serum ferritin values using peripheral venous blood samples obtained at admission were reviewed. In this NICU, serum ferritin is measured as a routine blood test for newborn infants who are admitted during the daytime or weekdays using the latex immunoturbidimetric assay (LABOSPECT 008, HITACHI, Tokyo, Japan). A total of 927 newborn infants were admitted to the NICU between April 2015 and March 2017. Of these, 348 newborn infants were hospitalized 24 h after birth, and 211 newborn infants were hospitalized within 24 h of age but during the night-time, weekend, and holidays, when the assay of serum ferritin values was unavailable. Finally, 368 newborn infants were included in the study cohort (Fig. 1). Physiological variables were obtained from the medical record of newborn infants and their mothers, including maternal complications, gestational age at birth, birth weight, sex, birth location, delivery mode, findings from blood tests, types of treatments, and clinical morbidities during hospitalization.

### Data analysis

Serum ferritin values were normalized by transforming data into natural logarithms. Univariable linear regression analysis was first performed to estimate the crude effects of the clinical background on serum ferritin values, excluding those with the incidence of less than 10. Findings from the univariable analysis were shown without correcting for multiple comparisons because of its exploratory nature. Statistical significance was set at *p*-value < 0.01. We created a multivariable linear regression model to explain the ferritin level, which was adjusted for sex, gestational age at birth, and the z-score of birth weight. Up to four independent variables were chosen based on the findings from the univariate analysis when theoretically relevant.

## Supplementary Information

Supplementary Information.

## Data Availability

The datasets generated during and/or analyzed during the current study are available from the corresponding author on reasonable request.

## References

[1] Erdman JWJ, Macdonald I, Zeisel SH (2012). Present Knowledge in Nutrition.

[2] Winter WE, Bazydlo LA, Harris NS (2014). The molecular biology of human iron metabolism. Lab. Med..

[3] Zhang C (2014). Essential functions of iron-requiring proteins in DNA replication, repair and cell cycle control. Protein Cell.

[4] Sangkhae V, Nemeth E (2019). Placental iron transport: The mechanism and regulatory circuits. Free Radic. Biol. Med..

[5] Rishi G, Subramaniam VN (2017). Biosci. Rep..

[6] Papazisis G (2008). Deferoxamine decreases the excitatory amino acid levels and improves the histological outcome in the hippocampus of neonatal rats after hypoxia-ischemia. Pharmacol. Res..

[7] Kyu HH (2016). Global and national burden of diseases and injuries among children and adolescents between 1990 and 2013: Findings from the global burden of disease 2013 study. JAMA Pediatr..

[8] Doom JR, Georgieff MK (2014). Striking while the iron is hot: Understanding the biological and neurodevelopmental effects of iron deficiency to optimize intervention in early childhood. Curr. Pediatr. Rep..

[9] Hoofnagle AN (2017). Harmonization of blood-based indicators of iron status: Making the hard work matter. Am. J. Clin. Nutr..

[10] Anderson GJ, Frazer DM (2017). Current understanding of iron homeostasis. Am. J. Clin. Nutr..

[11] Ganz T (2019). Erythropoietic regulators of iron metabolism. Free Radic. Biol. Med..

[12] Daru J (2017). Serum ferritin as an indicator of iron status: What do we need to know?. Am. J. Clin. Nutr..

[13] Beard J, deRegnier R-A, Shaw MD, Rao R, Georgieff M (2007). Diagnosis of Iron deficiency in infants. Lab. Med..

[14] Baynes RD (1996). Assessment of iron status. Clin. Biochem..

[15] Arosio P, Ingrassia R, Cavadini P (2009). Ferritins: a family of molecules for iron storage, antioxidation and more. Biochim. Biophys. Acta.

[16] Knovich MA, Storey JA, Coffman LG, Torti SV, Torti FM (2009). Ferritin for the clinician. Blood Rev..

[17] Arosio P, Elia L, Poli M (2017). Ferritin, cellular iron storage and regulation. IUBMB Life.

[18] Kon N (2010). Association between iron status and neurodevelopmental outcomes among VLBW infants. Brain Dev..

[19] Choudhury V (2015). Latent iron deficiency at birth influences auditory neural maturation in late preterm and term infants. Am. J. Clin. Nutr..

[20] Amin SB, Orlando M, Wang H (2013). Latent iron deficiency in utero is associated with abnormal auditory neural myelination in ≥ 35 weeks gestational age infants. J. Pediatr..

[21] Tonial CT (2017). Cardiac dysfunction and ferritin as early markers of severity in pediatric sepsis. J. Pediatr. (Rio J.).

[22] Schipper HM (1822). Neurodegeneration with brain iron accumulation—Clinical syndromes and neuroimaging. Biochim. Biophys. Acta.

[23] Ochiai M (2017). An elevation of serum ferritin level might increase clinical risk for the persistence of patent ductus arteriosus, sepsis and bronchopulmonary dysplasia in erythropoietin-treated very-low-birth-weight infants. Neonatology.

[24] Inoue H (2013). Serum neutrophil gelatinase-associated lipocalin as a predictor of the development of bronchopulmonary dysplasia in preterm infants. Early Hum. Dev..

[25] Ganz T (2019). Anemia of Inflammation. N. Engl. J. Med..

[26] Georgieff MK (2020). Iron deficiency in pregnancy. Am. J. Obstet. Gynecol..

[27] Sonoda M (2020). Prognostic factors for survival of herpes simplex virus-associated hemophagocytic lymphohistiocytosis. Int. J. Hematol..

[28] Nishitani M (2021). Hyperferritinemia: A diagnostic marker for disseminated neonatal herpes simplex virus infection?. Pediatr. Ann..

[29] Feldman AG, Whitington PF (2013). Neonatal hemochromatosis. J. Clin. Exp. Hepatol..

[30] Lai KC, Lorch SA (2022). Healthcare costs of major morbidities associated with prematurity in US children's hospitals. J. Pediatr..

[31] Celik HT, Yurdakok M, Korkmaz A, Yigit S (2015). Serum prohepcidin levels in premature newborns with oxygen radical diseases. J. Matern. Fetal Neonatal Med..

[32] Klevebro S (2016). Cohort study of growth patterns by gestational age in preterm infants developing morbidity. BMJ Open.

[33] Siddappa AM, Rao R, Long JD, Widness JA, Georgieff MK (2007). The assessment of newborn iron stores at birth: A review of the literature and standards for ferritin concentrations. Neonatology.

[34] Lorenz L, Peter A, Poets CF, Franz AR (2013). A review of cord blood concentrations of iron status parameters to define reference ranges for preterm infants. Neonatology.

[35] Siimes AS, Siimes MA (1986). Changes in the concentration of ferritin in the serum during fetal life in singletons and twins. Early Hum. Dev..

[36] Griffin IJ, Reid MM, McCormick KP, Cooke RJ (2002). Zinc protoporphyrin/haem ratio and plasma ferritin in preterm infants. Arch. Dis. Child. Fetal Neonatal Ed..

[37] Ru Y (2018). Predictors of anemia and iron status at birth in neonates born to women carrying multiple fetuses. Pediatr. Res..

[38] Basu S, Kumar D, Anupurba S, Verma A, Kumar A (2018). Effect of maternal iron deficiency anemia on fetal neural development. J. Perinatol..

[39] Meberg A, Hågå P, Sande H, Foss OP (1979). Smoking during pregnancy–hematological observations in the newborn. Acta Paediatr. Scand..

[40] Chockalingam UM, Murphy E, Ophoven JC, Weisdorf SA, Georgieff MK (1987). Cord transferrin and ferritin values in newborn infants at risk for prenatal uteroplacental insufficiency and chronic hypoxia. J. Pediatr..

[41] Rice L, Alfrey CP (2005). The negative regulation of red cell mass by neocytolysis: physiologic and pathophysiologic manifestations. Cell. Physiol. Biochem..

[42] Kim H-B (2021). Changes in hepcidin levels in an animal model of anemia of chronic inflammation: mechanistic insights related to iron supplementation and hepcidin regulation. Oxid. Med. Cell. Longev..

[43] Ozkan AK, Yemisci OU, Saracgil Cosar SN, Oztop P, Turhan N (2013). Can high-sensitivity C-reactive protein and ferritin predict functional outcome in acute ischemic stroke? A prospective study. Top. Stroke Rehabil..

[44] Ding H (2011). Hepcidin is involved in iron regulation in the ischemic brain. PLoS ONE.

[45] Azab SF (2016). Serum Hepcidin levels in childhood-onset ischemic stroke: a case-control study. Medicine (Baltimore).

