# Supplementary material for: Control variables of serum ferritin concentrations in hospitalized newborn infants: an observational study
Source: Sci Rep. 2023 May 24;13:8424. doi: 10.1038/s41598-023-35404-0 (PMC10209058; doi:10.1038/s41598-023-35404-0)
Supplement: Supplementary file 1 — Supplementary Information. [file 41598_2023_35404_MOESM1_ESM.docx]

**Supplementary File**

**Title**

Control variables of serum ferritin concentrations in hospitalized newborn infants: an observational study

**Authors**

Tadashi Hisano^1,2^, Junichiro Okada^2^, Kennosuke Tsuda^1^, Sachiko Iwata^1^, Shinji Saitoh^1^, and Osuke Iwata^1*^

**Affiliations**

^1^Center for Human Development and Family Science, Department of Neonatology and Pediatrics, Nagoya City University Graduate School of Medical Sciences, Nagoya, Aichi, Japan.

^2^Division of Neonatology, St. Mary's Hospital, Fukuoka, Japan.

***Correspondence and requests for reprints:**

Dr Osuke Iwata, Center for Human Development and Family Science

Department of Pediatrics and Neonatology, Nagoya City University Graduate School of Medical Sciences, Aichi, Nagoya 467-8601, Japan

Tel: +81-52-853-8246 Fax: (+81) 52-842-3449 E-mail: o.iwata@med.nagoya-cu.ac.jp

Online Supplementary Table 1: Serum ferritin levels

| All infants (n=368) | 149 [81.3, 236] |
| --- | --- |
| Gestational age ≤ 33 weeks (n=65) | 103 [62.0, 200] |
| 33 weeks < Gestational age ≤ 36 weeks (n=137) | 112 [69.0, 175] |
| 36 weeks < Gestational age (n=166) | 189 [122, 303] |

Values are shown as median [inter-quartile ranges].

Online Supplementary Table 2: Alternative multivariable models to predict serum ferritin levels at birth

A

|  | Regression coefficient | | | P-value |
| --- | --- | --- | --- | --- |
| Variables | Mean | 95% confidence interval | |  |
|  |  | Lower | Upper |  |
| Hypertensive disorders in pregnancy | -0.486 | -0.776 | -0.196 | 0.001 |
| Male sex | 0.081 | -0.112 | 0.274 | 0.411 |
| Z-score of birth weight | 0.262 | 0.162 | 0.362 | <0.001 |
| Lactate dehydrogenase (per 10^2^IU/L) | 0.077 | 0.055 | 0.099 | <0.001 |
| Hemoglobin (per g/dL) | -0.074 | -0.108 | -0.040 | <0.001 |
| pH | -1.390 | -2.330 | -0.448 | 0.004 |

B

| Variables | Regression coefficient | | | P-value |
| --- | --- | --- | --- | --- |
|  | Mean | 95% confidence interval | |  |
|  |  | Lower | Upper |  |
| Male sex | 0.036 | -0.112 | 0.274 | 0.411 |
| Birth weight (per 1000g) | 0.236 | 0.259 | 0.581 | <0.001 |
| Lactate dehydrogenase (per 10^2^IU/L) | 0.303 | 0.055 | 0.099 | <0.001 |
| Hemoglobin (per g/dL) | -0.190 | -0.108 | -0.040 | <0.001 |
| pH | -0.130 | -2.330 | -0.448 | 0.004 |
| Hypertensive disorders in pregnancy | -0.145 | -0.776 | -0.196 | 0.001 |

C

| Variables | Regression coefficient | | | P-value |
| --- | --- | --- | --- | --- |
|  | Mean | 95% confidence interval | |  |
|  |  | Lower | Upper |  |
| Male sex | 0.032 | -0.122 | 0.266 | 0.465 |
| Gestational age (per week) | 0.063 | -0.029 | 0.079 | 0.356 |
| Birth weight (per 1000g) | 0.187 | 0.088 | 0.579 | 0.008 |
| Lactate dehydrogenase (per 10^2^IU/L) | 0.298 | 0.053 | 0.098 | <0.001 |
| Hemoglobin (per g/dL) | -0.196 | -0.111 | -0.042 | <0.001 |
| pH | -0.127 | -2.305 | -0.421 | 0.005 |
| Hypertensive disorders in pregnancy | -0.146 | -0.779 | -0.198 | 0.001 |

D

| Variables | Regression coefficient | | | P-value |
| --- | --- | --- | --- | --- |
|  | Mean | 95% confidence interval | |  |
|  |  | Lower | Upper |  |
| Male sex | 0.031 | -0.163 | 0.224 | 0.754 |
| Gestational age (per week) | 0.080 | 0.045 | 0.116 | <0.001 |
| Lactate dehydrogenase (per 10^2^IU/L) | 0.075 | 0.053 | 0.098 | <0.001 |
| Hemoglobin (per g/dL) | -0.086 | -0.120 | -0.052 | <0.001 |
| pH | -1.460 | -2.410 | -0.513 | 0.003 |
| Hypertensive disorders in pregnancy | -0.537 | -0.828 | -0.247 | <0.001 |
